# H7 Coil Deep Repetitive Transcranial Magnetic Stimulation in Patients With Obsessive-Compulsive Disorder: Do Factors Like Age, Sex, and Medication Affect Clinical Outcomes?

**DOI:** 10.7759/cureus.91447

**Published:** 2025-09-01

**Authors:** Aswin Kumar Mudunuru, Lalitha Jahnavi, MS Reddy, Swetha Reddy, Kartik Valipay, Balaji Sainath A, Chandresh N, Madhiha M, Bhavana Allam, Sailaja Bomma

**Affiliations:** 1 Non-invasive Brain Stimulation, Asha Neuromodulation Clinics, Hyderabad, IND; 2 Psychology, Asha Neuromodulation Clinics, Hyderabad, IND; 3 Psychiatry, Asha Hospital, Hyderabad, IND; 4 Psychiatry, Asha Neuromodulation Clinics, Hyderabad, IND; 5 Psychiatry, Asha Neuromodulation Clinics, Bengaluru, IND; 6 Psychiatry and Behavioral Sciences, Asha Neuromodulation Clinics, Hyderabad, IND

**Keywords:** h7 coil, obsessive-compulsive disorder (ocd), safety and efficacy, transcranial magnetic stimulation (tms), ybocs

## Abstract

Background

Deep repetitive transcranial magnetic stimulation (DTMS), using the H7 coil, is an approved treatment for obsessive-compulsive disorder (OCD) by the United States Food and Drug Administration (US-FDA). The clinical efficacy and safety of accelerated regimens of DTMS have been previously studied extensively. This study reiterates the efficacy of accelerated DTMS (aDTMS) and examines the influence of factors such as age, sex, and medication on clinical outcomes in the Indian population.

Methods

A retrospective data analysis of 378 OCD patients was conducted. aDTMS sessions were given as a combination of intermittent theta burst stimulation (iTBS) and high-frequency stimulation (HFS) over eight days, with two sessions per day. The primary outcome measure was the Yale-Brown Obsessive-Compulsive Scale (YBOCS) scores, assessed before and after 15 sessions of aDTMS.

Results

A significant mean reduction in Day 8 YBOCS scores (11.12 ± 7.46, Cohen’s d = 2.2, p < 0.0001), with an overall response rate of 64.72% and a remission rate of 46.12%, was observed (N = 378). The study also found that patients of both sexes, aged 30-44 years, showed the highest response to treatment. Women with severe to extreme disease, and menopausal women, did not respond better than men. The number of drugs had no significant effect on the outcome.

Conclusions

The findings support the potential of aDTMS as an effective adjunctive treatment for OCD, highlighting the interplay of various factors in determining clinical outcomes.

## Introduction

Obsessive-compulsive disorder (OCD) is a psychiatric disorder characterized by uncontrolled intrusive thoughts, called obsessions, followed by compulsive behaviors that are performed as a response to obsessions. OCD is ranked 10th among the most debilitating disorders by the WHO and is associated with time-consuming symptoms, like distressing repetitive thoughts and compulsive rituals, which can significantly disrupt functionality and quality of life in a patient [1]. With a lifetime prevalence of 1%-3%, OCD is often misdiagnosed and undertreated [2]. A substantial proportion of OCD patients remain unresponsive to the conventional first line of treatments, like cognitive behavioral therapy (CBT) and selective serotonin reuptake inhibitors (SSRIs), due to heterogeneous characterization in etiology and symptoms [2]. Additionally, in OCD, due to delayed diagnosis, patients often fail to achieve complete symptomatic remission. Although existing pharmacological and therapeutic interventions succeed in attaining initial brief symptom relief, a few patients fail to maintain this improvement, thereby leading to weakened compliance and reduced treatment adherence [3,4].

Non-invasive brain stimulation (NIBS) is a promising adjunctive treatment for OCD. NIBS targets specific cortical areas, like the dorsomedial prefrontal cortex (dmPFC), anterior cingulate cortex (ACC), supplementary motor area (SMA), etc., resulting in better treatment outcomes among OCD patients [5]. The United States Food and Drug Administration (US-FDA) approved the use of the H7 coil in 2018 and the Cool D-B80 coil in 2022, using different treatment parameters, for the treatment of OCD [6]. Deep repetitive transcranial magnetic stimulation (DTMS) with the H7 coil can stimulate the deeper and broader areas of the cortical network and produce better clinical outcomes [7,8].

The therapeutic effects of TMS can be classified as classical and non-classical. Modulation in neurotransmitter concentrations, synaptic plasticity via long-term potentiation, and long-term depression are the classical effects. TMS has also been shown to influence dendritic growth and sprouting through the production of neurotrophic factors, like brain-derived neurotrophic factor [9]. The non-classical effects of TMS are related to the biophysical effects of magnetic fields, including quantum effects, magnetic spin effects, genetic magnetoreception, and macromolecular effects [9,10]. A theoretical plausibility exists that these effects could be magnified by producing stronger and deeper TMS-induced electric fields (E-fields) in the brain to facilitate modulation of neuronal ensembles over a wide area, as well as activation of projection neurons. There are multiple large-scale studies supporting the use of DTMS as an adjuvant to pharmacotherapy, showing promising response and remission rates while following standard treatment protocols [11,12].

Long-term potentiation-like effects can be produced by TMS using high-frequency stimulation (HFS) at a 20 Hz train frequency or intermittent theta burst stimulation (iTBS), which is a combination of 5 Hz and 50 Hz. Accelerated TMS treatment regimens, delivering more than a single session per day (as high as 10 sessions), have been tried in depression [13,14]. Accelerated DTMS (aDTMS) treatment with the H7 coil has been previously tested in observational studies in a real-world setting and has shown a positive impact on outcomes [11,15]. However, a detailed understanding of the effects of factors like age, sex, menopausal status, and medication remains to be explored. 

The objective of the present study was to assess the efficacy of aDTMS in OCD by measuring the Day 8 reduction of the Yale-Brown Obsessive-Compulsive Scale (YBOCS), and to explore the impact of a few factors on this outcome.

## Materials and methods

In the current retrospective observational study, clinical data were collected from all outpatients who were diagnosed with OCD according to DSM-5 guidelines and visited the Neuromodulation clinics during the period from April 2022 to March 2025 (36 months). A total of 378 patients were selected using consecutive sampling, who received the aDTMS treatment during this period. The patient group was a mixed population with different drug prescriptions and durations of illness, and with or without medical comorbidities, such as hypertension, diabetes mellitus, hypothyroidism, etc. Prior to treatment, all patients had given written informed consent to use their demographic and treatment details for research purposes. Parent or guardian consent was also taken in the case of adolescent patients. All patients went through a systematic screening procedure to assess their compatibility with the electromagnetic fields of the TMS. Patients belonging to both sexes and in the age group of 12-89 years were included. The presence of ferromagnetic materials in the head and neck region, history of traumatic brain injury, presence of pacemakers, cochlear implants, or other implanted devices were the exclusion criteria [16]. Patients with psychosis and severe substance use disorders were excluded. Clinical data of all the patients satisfying the inclusion criteria were considered for analysis. 

The primary outcome measures were the YBOCS scores, measured after completion of 15 sessions (Day 8), and compared to baseline Day 1 scores [17]. The response rate in OCD was calculated as a >35% reduction in YBOCS score on Day 8 [15]. The remission rate was calculated as a YBOCS < 12 on Day 8. All rating scales were administered by psychologists before (Day 1) and after the treatment (Day 8). The post-DTMS score was evaluated by another psychologist to rule out operator bias.

The device used was BrainsWay’s 104 deep TMS system (BrainsWay Ltd., Jerusalem, Israel), fitted with the H7 coil. The OCD treatment protocol extended over 15 sessions in eight days, with the aDTMS regimen administered as 15 total sessions, given as two per day for about 7.5 consecutive days. Each session consisted of an iTBS preceding every HFS. (a) The iTBS600 stimulation, with a standard 5Hz-50Hz combination of train and burst frequencies, respectively, was given at 80% of resting motor threshold (RMT) as a priming session, with the importance of iTBS600 as a priming session studied previously [18]. (b) The HFS, with 50 two-second trains of 20 Hz and a 20-second interval, was given at 100% of RMT, delivering 2000 pulses. This combination of iTBS600 + HFS20 consisted of 2600 pulses. Fifteen such sessions, covering 7.5 days at a frequency of two sessions per day, constituted the OCD treatment, delivering a total of 39,000 pulses. No specific behavioral techniques were employed during the sessions, except for supportive counselling as needed

The patients were classified based on age, sex, disease severity, and number of drugs. Special emphasis has been placed on the effect of menopause on the clinical outcome, and the side effect profile has been explored.

Statistical analysis

The preliminary data were entered into MS Excel (Microsoft® Corp., Redmond, WA, USA); further statistical analysis was performed using GraphPad Prism software (GraphPad Software, Inc., San Diego, CA, USA). A paired sample t-test was performed to test the significance of the mean reduction in YBOCS scores. Cohen’s d was calculated to estimate the effect size. Multiple regression analysis was done to determine the effect of individual factors like age, sex, and number of drugs on the difference in YBOCS from Day 1 to Day 8.

## Results

The total number of patients included for analysis is 378, with a mean age of 34.96 ± 12.27 years. Of the 378 patients, 178 were female (mean age 36.12 ± 13.34 years), and 200 were male (mean age 33.99 ± 11.20 years). All patients were divided into different age groups representing adolescents, early and late adults, middle-aged, and older adults. About 75% of the patients belonged to the 18-44 years age group, mostly men. Sixteen patients were adolescents, with a mean age of 15 years. Selecting adolescent patients for dTMS required satisfaction of a few criteria, such as head size suitable for the H7 coil and absence of a family history of seizures (Table 1).

**Table 1:** Age-wise distribution of patients expressed as number (N)

Age group (in years)	Description	Mean age (Mean ± SD)	Female subjects (N)	Male subjects (N)	Total (N)/% of total	% of total
12-17	Adolescents	15.81 ± 1.33	12	4	16	5
18-29	Early adults	24.62 ± 3.33	48	82	130	34
30-44	Late adults	36.03 ± 3.87	73	78	151	40
45-60	Middle age	51.05 ± 4.73	36	22	58	15
61-89	Old age	66.85 ± 5.88	9	14	23	6
	Total		178	200	378	100

**Figure 1:**
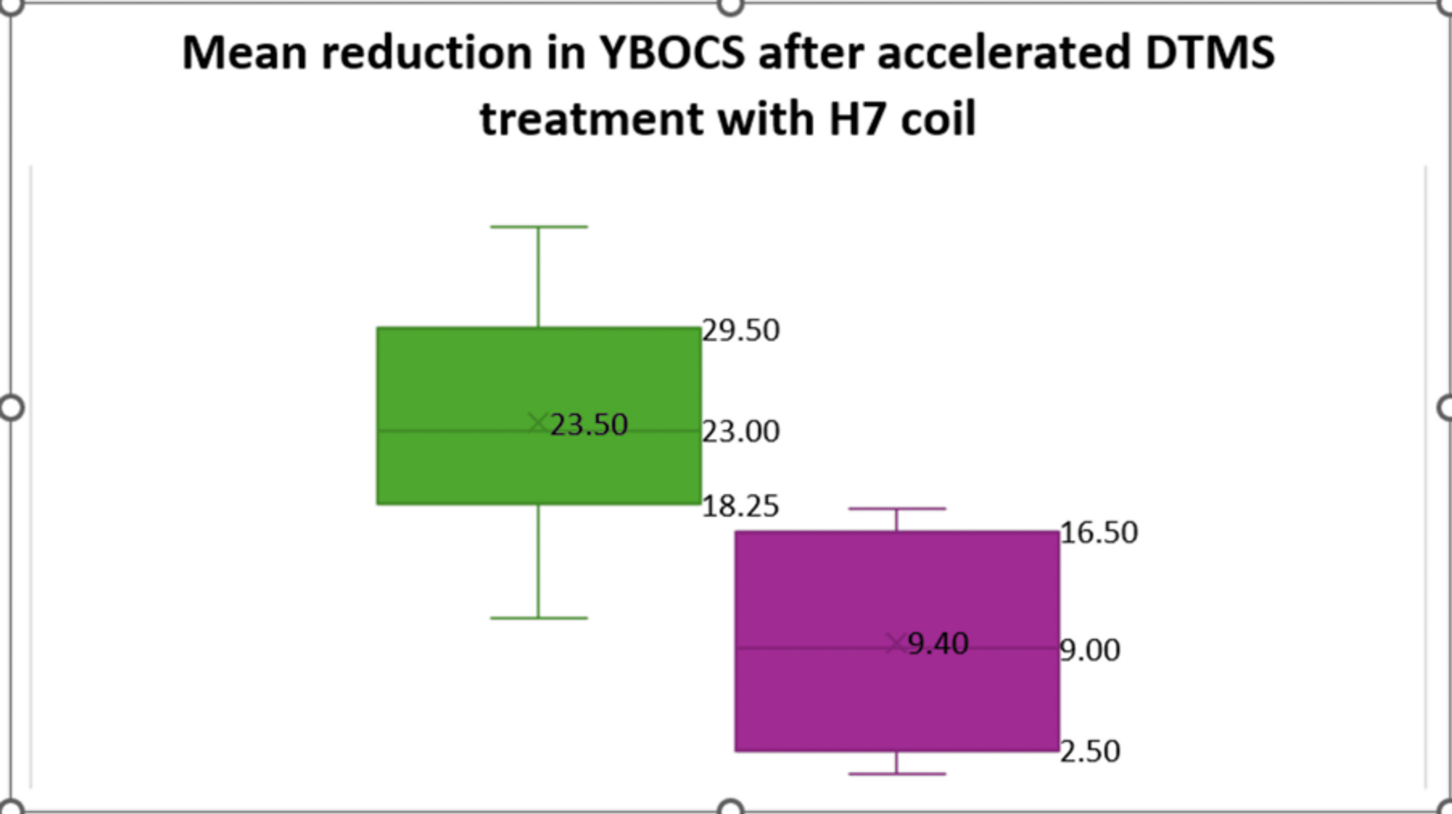
Boxplot showing mean reduction in YBOCS scores on day 8 after 15 sessions of DTMS with H7 coil YBOCS, Yale-Brown Obsessive-Compulsive Scale; DTMS, Deep Repetitive Transcranial Magnetic Stimulation

When classified based on disease severity, it was found that about 62% of the patients who received dTMS had a moderate to severe form of the disease, almost equally divided between male and female patients (Table 2). Accordingly, the response rate in this group was about 60%, compared to the overall response rate of 64.72% (Table 3). Although the overall mean reduction in the YBOCS score on Day 8 was 13.68 ± 8.22 - slightly higher in females (14.94 ± 8.80) compared to males (13.15 ± 6.93) (p = 0.243, non-significant, d = 0.34) - the response rates were slightly higher in men compared to women (p = 0.04), which was considered significant after correction for multiple comparisons (Table 4 and Figure 1).

**Table 2:** Age-wise distribution of patients expressed as number (N)

Age group (in years)	Description	Day 1-Day 8 YBOCS	% reduction in YBOCS	No. of patients who showed >35% reduction	Total no. of patients in the age group	Response rate (%)
		Mean ± SD				
12-17	Adolescents	11.62 ± 6.63	46.23 ± 20.87	10	16	62
18-29	Early adults	13.19 ± 9.08	54.52 ± 32.22	81	130	62
30-44	Late adults	14.67 ± 9.48	62.77 ± 31.42	105	151	69
45-60	Middle age	12.67 ± 8.16	53.68 ± 31.50	33	58	57
61-89	Old age	15.39 ± 10.24	66.84 ± 35.25	14	23	61
	Total				378	

**Table 3:** Disease severity-wise and sex-wise mean reductions in YBOCS in all OCD patients after 15 DTMS sessions YBOCS, Yale-Brown Obsessive-Compulsive Scale; OCD, Obsessive-Compulsive Disorder; DTMS, Deep Repetitive Transcranial Magnetic Stimulation

Measure	YBOCS range	Day 1 YBOCS (Y1)		Day 8 YBOCS (Y8)		Diff. in YBOCS (Y8-Y1)		% reduction in YBOCS score	
		Mean	SD	Mean	SD	Mean	SD	Mean	SD
All patients (n = 378)	Overall	24.81	8.19	13.68	8.22	11.12	7.46	45.81	25.56
	Mild (n = 68)	11.97	2.33	5.42	3.06	6.54	2.37	55.88	19.74
										Moderate (n = 115)	19.07	2.07	10.52	5.29	8.55	5.35	44.58	27.39
	Severe (n = 119)	27.33	2.27	15.8	7.54	11.53	6.77	42.86	25.77
	Extreme (n = 76)	34.92	2.51	20.25	8.59	14.66	8.15	42.25	23.94
	Only male patients (n = 200)	Overall (n = 200)	24.36	7.65	13.15	6.93	11.2	6.79	46.11	22.44
Mild (n = 36)											12.52	1.87	6.36	2.71	6.89	2.51	56.55	22.64
		Moderate (n = 60)	18.91	1.88	9.82	5.06	9.08	5.42	47.39	27.59								
Severe (n = 64)											24.36	2.33	12.6	7.27	12.06	6.45	45.13	24.83
		Extreme (n = 40)	34.63	2.53	17.13	8.04	17.5	7.59	50.85	23.15								
Only female patients (n = 178)											Overall (n = 178)	25.33	8.76	14.94	8.8	10.38	7.46	42.4	25.31
		Mild (n = 32)	11.31	2.56	5.18	2.63	6.12	2.05	55.08	15.11
											Moderate (n = 55)	19.25	2.25	11.28	5.43	7.96	5.22	41.5	26.82
		Severe (n = 55)	27.58	2.14	16.71	7.05	10.87	6.69	39.64	24.17									
											Extreme (n = 36)	35.13	2.48	22.62	8.23	12.51	7.91	35.72	22.43

**Table 4:** Response rate and remission rates after 15 sessions of aDTMS with H7 coil in OCD patients aDTMS, Accelerated Deep Repetitive Transcranial Magnetic Stimulation; OCD, Obsessive-Compulsive Disorder

Criterion	All patients (N = 378)		Only male patients (N = 200)		Only female patients (N = 178)	
	Response rate % (N)	Remission rate % (N)	Response rate % (N)	Remission rate % (N)	Response rate % (N)	Remission rate % (N)
Overall	64.72 (378)	46.12 (378)	67.14 (200)	52.14 (200)	59.32 (178)	38.90 (178)
Mild disease	82 (68)	97.14 (68)	84.21 (36)	47.36 (36)	76.47 (32)	100 (32)
Moderate disease	65.6 (115)	62.6 (115)	74.85 (60)	57.14 (60)	59.37 (55)	53.12 (55)
Severe disease	54 (119)	30 (119)	55.73 (64)	34.42 (64)	56.41 (55)	28.21 (55)
Extreme disease	64.7 (76)	15.68 (76)	81.81 (40)	72.72 (40)	51.72 (36)	6.89 (36)

About 20% of the female patients, who were postmenopausal for an average period of 14 ± 3.21 years and received aDTMS treatment, showed about a 46% response - significantly lower compared to the perimenopausal group (p = 0.031, d = 0.36) and the reproductive group (p = 0.020, d = 0.48) (Table 5).

**Table 5:** Effect of menopause on response to treatment YBOCS, Yale-Brown Obsessive-Compulsive Scale

Group	Age (Mean ± SD)	Day1 YBOCS (Mean ± SD)	Day 8 YBOCS (Mean ± SD)	Diff. in YBOCS (Mean ± SD)	% response (Mean ± SD)
Reproductive (N = 71)	27.82 ± 4.91	23.30 ± 5.97	13.67 ± 8.09	13.67 ± 8.46	60.72 ± 34.47
Perimenopausal (N = 55)	40.78 ± 3.08	25.98 ± 10.06	15.32 ± 8.67	13.98 ± 9.77	59.65 ± 30.41
Menopausal (N = 36)	55.81 ± 7.12	24.10 ± 10.37	18.00 ± 9.20	9.25 ± 9.75	45.68 ± 34.69

When the patients were classified into groups based on the number of drugs prescribed, the majority of prescriptions for the recruited OCD patients consisted of SRIs, which were present in one- to three-drug regimens. The four- to five-drug users were additionally prescribed a benzodiazepine (BZD), an antipsychotic, or other medications. The mean reduction in YBOCS was slightly higher in the three- to four-drug users compared to the one- to two-drug users, but this difference was not statistically significant (p = 0.5, d = 0.77) (Table 6).

**Table 6:** Distribution of mean reductions in YBOCS according to number of drugs YBOCS, Yale-Brown Obsessive Compulsive Scale

Number of drugs	Number of patients (n)	Day 1 YBOCS		Day 8 YBOCS		Difference in YBOCS score		% response	
		Mean	SD	Mean	SD	Mean	SD	Mean	SD
1 drug	81	24.75	8.26	15.10	8.29	9.64	5.28	42.02	21.43
2 drugs	90	24.75	8.13	15.33	8.70	9.41	5.59	41.29	24.51
3 drugs	99	23.93	7.46	12.20	7.71	11.72	7.33	49.63	25.56
4 drugs	65	25.9	9.25	13.7	7.32	12.2	9.74	44.94	25.82
5 drugs	40	27.95	7.50	14.08	7.0	13.87	5.64	50.69	17.24

Multiple regression analysis with age, sex, and number of drugs as predictors of Day 8 reduction in YBOCS score showed a very weak collective effect (F(1, 331) = 9.19, p = 0.003, R² = 0.03, or 3%) and a weak correlation between predicted and observed YBOCS scores (R = 0.16).

**Figure 2:**
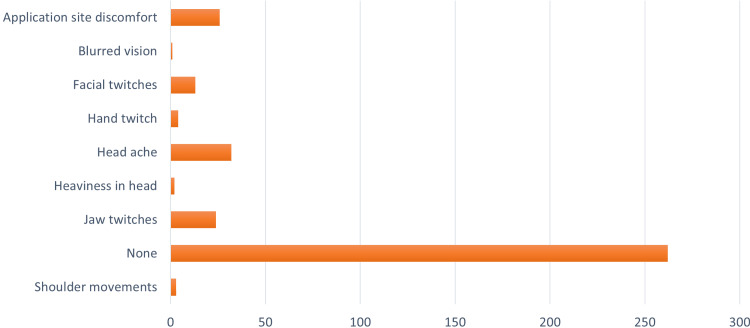
Clustered bar chart showing the side effects noticed during the DTMS treatment DTMS, Deep Transcranial Magnetic Stimulation

About 69% of the patients reported no side effects during the treatment. There were no seizures or other serious adverse events. Application site discomfort, headache, and jaw twitches were the most common side effects, observed in about 2% of the total patients. Blurred vision was reported transiently by a 39-year-old male during the 14th session. This could possibly be due to dTMS (Figure 2).

## Discussion

Former research suggests that there is a positive impact of DTMS on the recovery from OCD [11,12,19,20]. The current observational study is in congruence with this, even when given in an accelerated fashion. The study group consisted of a heterogeneous population with multiple types of obsessions, ranging from moderate to severe disease severity. The number of drugs being used and the duration of illness were also variable. However, the aDTMS regimen was uniform across all patients. A detailed pre-TMS safety questionnaire helped rule out unstable and unfit patients for DTMS treatment. Unstable patients with uncontrolled metabolic, cardiovascular, or any endocrine disorders, electrolyte disturbances, traumatic brain injury, and ferromagnetic incompatibility were excluded from the treatment.

The overall response rate in OCD after 15 DTMS sessions was observed to be 64.72%, and remission from the disease was seen in 46.12% of the patients. This is in agreement with previous observational studies [15]. The authors believe that a 50% reduction in YBOCS on Day 8 is an encouraging progress, viewed from a clinical perspective. Pharmacotherapy was maintained as usual during the treatment. Contrary to previous studies, our aDTMS regimen did not employ provocation techniques due to the lessons learned from our clinical experience. Provocation suits a subset of OCD patients, but generalizing it is known to produce greater anxiety and poor compliance in our population, probably due to sociocultural factors. The study demonstrates that good clinical improvement is possible even in the absence of provocation. The compliance of the patients with the accelerated regimen was good. In fact, our clinical experience shows that many patients opt for accelerated regimens due to operational ease. Achieving early functional recovery in chronic severe forms of OCD is encouraging, reflected in an average 11-point reduction on Day 8 YBOCS. The current observational study further explored the possible effects of a few factors on this outcome. Our analysis does not establish a causal relationship between these factors and the outcome, but it helps to understand them from a clinical perspective.

Age of the patients

Although the most common age of onset of OCD is about 18-29, 74% of the patients were in the age group of 18-44 years (Tables 1-2). Possible factors could include a delay in psychiatric consultation, a delay due to multiple drug trials before testing DTMS, or the patient's agreement to undergo DTMS treatment, indicating the need to create awareness about the safety and non-invasiveness of DTMS as an adjunct to standard pharmacotherapy. Furthermore, among the 74% of the adult population of patients, the majority (57%) were men, and 32% were women. The mean percentage reduction in YBOCS was greatest in the age group of 30-44 years, corresponding to 62.77 ± 31.42%. The response rate in this age group was also the highest, at 69%. It is worth noting that the H7 coil also performed better in geriatric patients. As a variable degree of cortical atrophy is expected in old age, the deeper and broader stimulation of DTMS helped achieve a good recovery in this age group [21,22].

Sex

The current study group consisted of almost equal proportions of males and females, 53% and 47%, respectively. Previous studies explain that the sex of the patient could influence clinical outcomes by altering neuronal plasticity [23]. Concurrently, the pathophysiology of OCD explains the greater prevalence in women [22]. The average percentage reduction in Day 8 YBOCS score was non-significantly different in men and women (p = 0.341). However, women with severe and extreme forms of the disease responded much less to the DTMS treatment compared to men (p = 0.001) (Table 3). The majority of patients with severe to extreme disease were in the age group of 30-44 years for females, as compared to 18-44 years for men. To summarize, men responded better than women in cases of severe to extreme forms of the disease.

Menopause

As seen from Table 5, perimenopausal and menopausal women had severe to extreme forms of OCD. After DTMS treatment, the average Day 8 reduction in the YBOCS score was about 9 points, compared to 14 points in the other groups.

Number of drugs

Table 6 depicts that three-drug users (n = 99) showed the lowest Day 8 YBOCS scores, almost close to remission, and had the maximum number of responders (YBOCS score < 35%). Although five-drug users showed a similar response, their number was very small (10%) compared with other groups. About 90% of three-drug users were taking two SRIs and one BZD. However, the slightly higher average YBOCS reduction seen in three- to four-drug users was not statistically significant. Probably, a larger sample size would elucidate drug-DTMS interactions. 

Limitations

The study has the following limitations: the sample included patients with multiple types of obsessions; a detailed evaluation could have been done on the response rates for different types of obsessions. Details about the prescription drugs and their dosages could have been studied against the response shown to aDTMS treatment. The study design, lack of a control group, and limited follow-up duration are other limitations of the study.

## Conclusions

Acceleration of DTMS treatment in OCD patients was safe and effective in all age groups of patients. Administration of 15 sessions over eight days produced significant reduction in the average YBOCS scores in all patients. Middle aged men belonging to all degrees of disease severity showed better response than women. Menopausal women showed significantly lower response compared to reproductive age women.
